# Histologic Features of Melanocytic Nevi Secondary to Melanoma Treatment With Immune Checkpoint Inhibitors

**DOI:** 10.7759/cureus.105392

**Published:** 2026-03-17

**Authors:** Nehaa Sohail, Ayaan Sohail, Sanaa Durrani, Jorge Bilbaoa, Cesar A Abbud, Brenda Simpson, Cynthia Reyes Barron

**Affiliations:** 1 Medical Education, Texas Tech University Health Sciences Center Paul L. Foster School of Medicine, El Paso, USA; 2 Medical Education, McGovern Medical School, The University of Texas Health Science Center, Houston, USA; 3 Medical Education, Texas Tech University Health Sciences Center El Paso Paul L. Foster School of Medicine, El Paso, USA; 4 Pathology and Laboratory Medicine, Pathology Professional Services, El Paso, USA; 5 Department of Dermatology, El Paso Dermatology, El Paso, USA

**Keywords:** cytotoxic t-lymphocyte-associated protein 4 inhibitors, immunotherapy, ipilimumab, melanocytic nevi, melanoma, nivolumab, programmed death-1 inhibitors

## Abstract

Programmed death-1 (PD-1) inhibitors are anticancer drugs that function as immune checkpoint blockers, targeting PD-1 proteins on the surface of cells to restore immune system activity against tumors. While cutaneous adverse events associated with immune checkpoint inhibitor therapy are well documented, this case highlights the reactive histopathologic changes that can occur in benign nevi during treatment. Here, we present the case of a 51-year-old Hispanic female with a history of melanoma on the chest who was seen in clinic for routine follow-up. Three suspicious pigmented lesions located on the right upper back, abdomen, and left shoulder were biopsied for further analysis. Histopathologic examination indicated an intradermal melanocytic nevus with irritation and inflammation, an inflamed, mildly dysplastic compound melanocytic nevus, and a compound melanocytic nevus with marked inflammation and no atypia. Additional clinical history revealed that the patient was receiving treatment for advanced melanoma with immune checkpoint inhibitors. Increased awareness of these features in melanocytic lesions associated with PD-1 and cytotoxic T-lymphocyte-associated protein 4 (CTLA-4) inhibitors may help with accurate diagnosis.

## Introduction

Programmed death-1 (PD-1) inhibitors are checkpoint inhibitor anticancer drugs that block the activity of PD-1 immune checkpoint proteins on the surface of cells. PD-1 binds to programmed death-ligand 1 (PD-L1), which is a protein on both normal and cancer cells. This binding acts like an off switch to keep T cells from attacking other cells within the body, suppressing the immune system and preventing autoimmune diseases [1]. Two checkpoint inhibitors, nivolumab and ipilimumab, are used as treatment for melanoma as early as Stage II and are preferred systemic therapy for metastatic disease (Stage III or greater). Various immune-related adverse effects (IRAEs) have been described in the literature, including regressive changes of melanocytic nevi [2-4]. The effects on melanocytic nevi may not cause significant morbidity; however, recognizing the potential histologic changes on biopsy may aid in accurate diagnosis, particularly in a patient population with a known history of melanoma. We describe the case of a patient being treated with nivolumab and ipilimumab for advanced melanoma, with biopsy of three melanocytic lesions demonstrating marked inflammation and reactive atypia on histology.

## Case presentation

**Figure 1:**
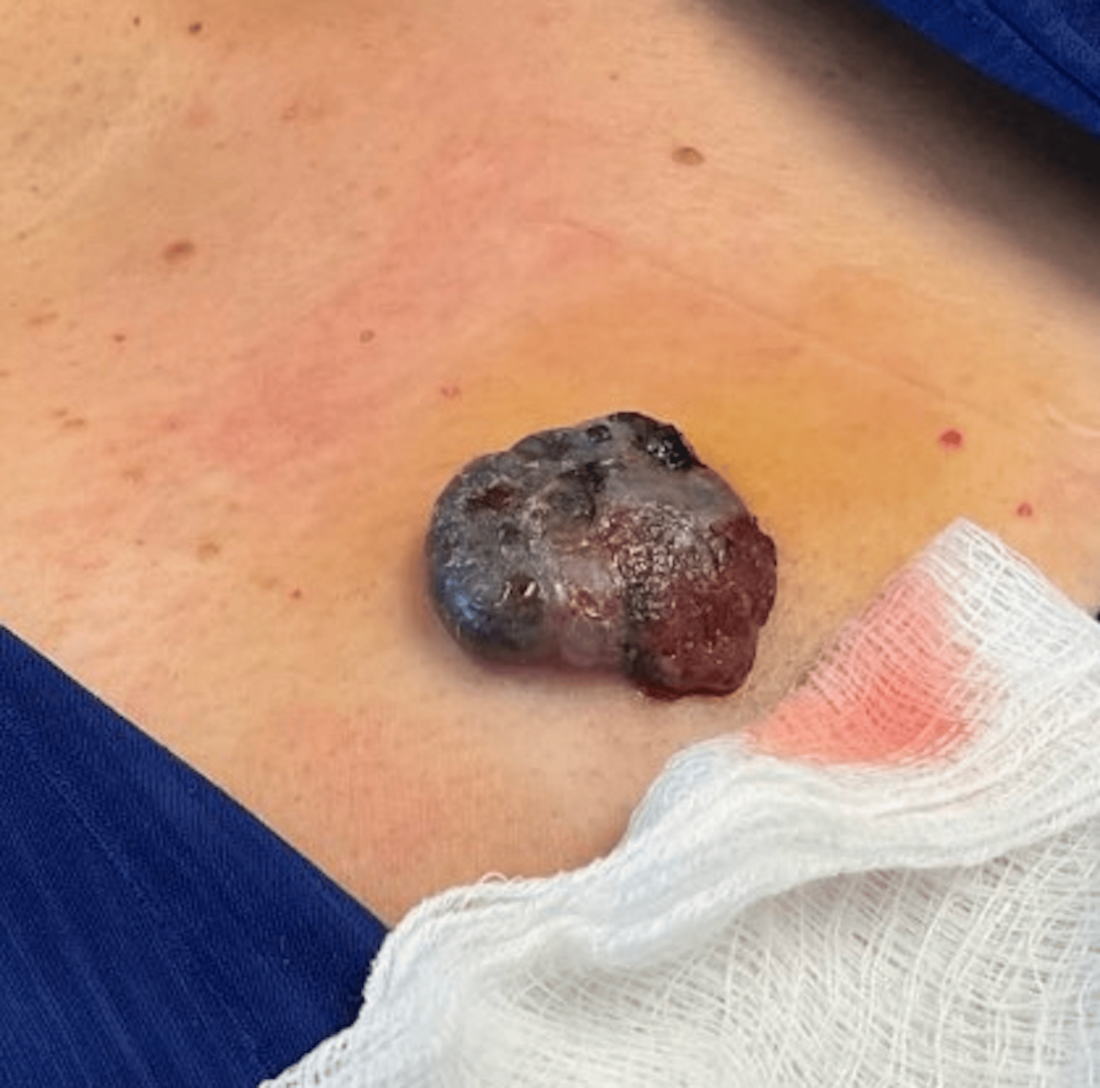
Ulcerated, exophytic pigmented nodule on the anterior chest wall demonstrating irregular borders, hemorrhagic crusting, and variegated coloration.

A 51-year-old Hispanic female was being followed in the dermatology clinic after being diagnosed with melanoma of the chest. On initial biopsy, the melanoma was staged at least pT2b with a depth of at least 1.7 mm and ulceration. Metastasis was subsequently identified, and she began treatment with nivolumab and ipilimumab immunotherapy. Almost seven months after the melanoma diagnosis, three unusual pigmented lesions were identified on skin examination on the right upper back, abdomen, and left shoulder (Figure 1). These were biopsied and submitted for pathology evaluation.

**Figure 2:**
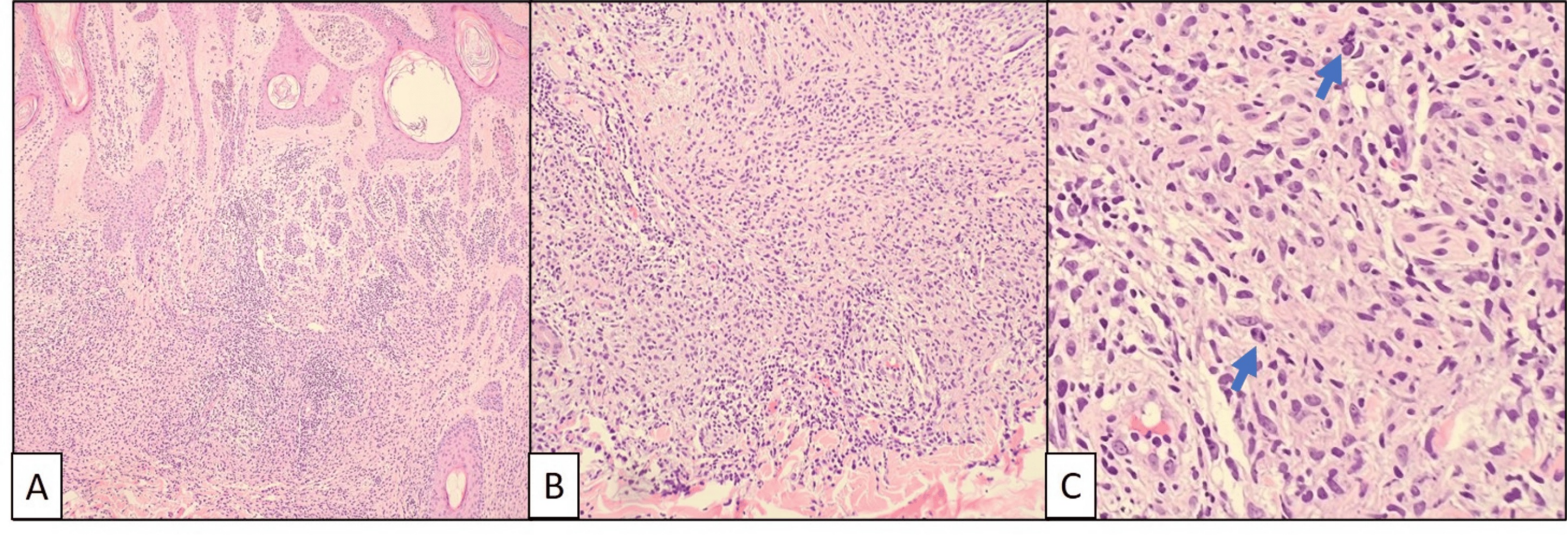
Hematoxylin and eosin-stained sections of the intradermal melanocytic nevus from the right upper back. (A) Epidermal changes including hyperkeratosis and acanthosis were noted. There was adequate maturation of melanocytes. (B) The lymphohistiocytic infiltrate was present throughout the melanocytic lesion. (C) Mitotic figures in the reticular dermis are indicated by blue arrows.

The lesion from the right upper back was diagnosed as an intradermal melanocytic nevus. Histology showed a cellular melanocytic proliferation in the dermis displaying adequate maturation with descent into the deep dermis (Figure 2). A marked lymphohistiocytic inflammatory infiltrate was present throughout the lesion, and mitotic activity was identified. A double stain for Melan-A and Ki-67 confirmed that proliferation was brisk within inflammatory cells but sparse in melanocytes. A stain for PRAME (Preferentially Expressed Antigen in Melanoma) was only focally positive, primarily in superficial melanocytes, and a stain for p16 was retained, both staining patterns supportive of a benign process.

**Figure 3:**
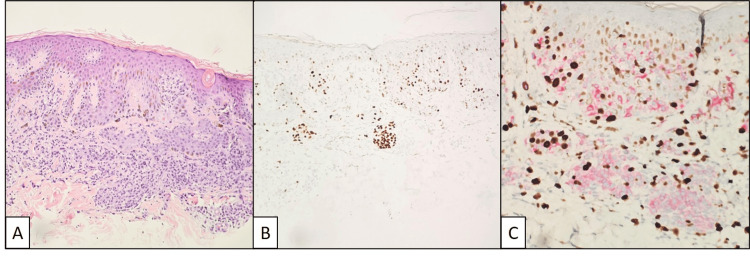
Mildly dysplastic compound melanocytic nevus from the abdomen. (A) Mild cytologic atypia and architectural disorder were noted on hematoxylin and eosin-stained sections. (B) SOX10 highlighting the epidermal and dermal components with no significant Pagetoid spread. (C) Melan-A (red) and Ki-67 (brown) double stain with dermal mitotic activity primarily limited to inflammatory cells.

The lesion from the abdomen was diagnosed as a compound melanocytic nevus with architectural disorder and mild cytologic atypia (dysplastic nevus) (Figure 3). The melanocytic proliferation was not sharply circumscribed, and although nests were predominant, there were scattered single cells along the dermal epidermal junction. Pagetoid cells were not identified. Cytologic atypia was considered mild and slightly greater than could be attributed to reactive changes alone. Like the lesion from the upper back, a marked lymphohistiocytic inflammatory infiltrate was also present throughout, with mitotic activity identified in inflammatory cells. Maturation was adequate; a stain for p16 was retained, and a stain for PRAME was negative.

**Figure 4:**
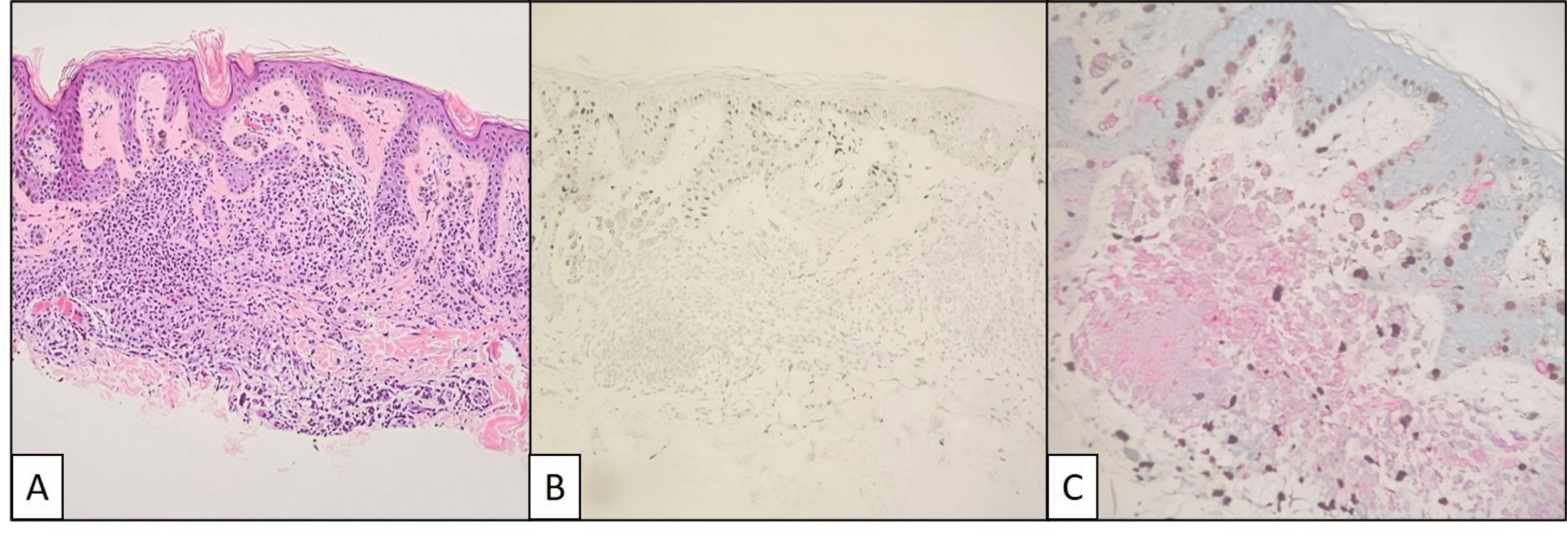
Compound melanocytic nevus from the left shoulder. (A) Hematoxylin and eosin-stained sections demonstrating marked lymphohistiocytic inflammation throughout the lesion. (B) Predominantly negative PRAME stain in melanocytes. (C) Melan-A (red) and Ki-67 (brown) double stain with dermal mitotic activity primarily limited to inflammatory cells.

The lesion from the left shoulder was diagnosed as a markedly inflamed compound melanocytic nevus with no dysplasia (Figure 4). This lesion was well circumscribed with melanocytes growing primarily in nests in the epidermis and displaying adequate maturation with descent into the dermis. The staining pattern was similar to the previous, with retention of p16 and negative PRAME. The brisk lymphohistiocytic inflammatory infiltrate and proliferation patterns were also similar to the others. The patient did not develop other adverse skin or non-skin reactions from immune checkpoint inhibitor therapy.

## Discussion

Immune checkpoint inhibition has become a widely used therapeutic mechanism for the treatment of malignancy and common autoimmune disorders [5,6]. This case presents a patient who was treated with PD-1 and CTLA-4 inhibitors for melanoma and developed cutaneous IRAEs. The history of melanoma with metastasis and presentation with atypical, pigmented lesions on skin examination raised concern that the additional pigmented lesions biopsied represented cutaneous metastases or additional primary melanomas. Careful histologic examination and immunohistochemistry were instrumental in excluding malignancy. Confirmation of the treatment history with PD-1 and CTLA-4 inhibitors and knowledge that these may result in reactive changes in benign nevi provided an explanation for the histologic findings observed.

Once a patient is diagnosed with melanoma, particularly with metastasis and aggressive features, close clinical follow-up is warranted. The threshold for biopsy for pigmented lesions lowers when the risk of metastasis and additional primary melanomas is higher. Patients with multiple melanocytic nevi and a previous melanoma diagnosis are also at greater risk for second primary melanomas [7]. The pigmented lesions biopsied after melanoma diagnosis in this patient displayed concerning features clinically, including irregularity and evolution.

Cutaneous changes caused by PD-1 inhibition are a relatively common side effect among patients with melanoma [1]. Specific changes that have been documented include vitiligo and halo nevus, also known as Sutton nevus [4]. The halo nevus is a melanocytic nevus that is clinically surrounded by depigmentation, resembling a halo [4]. Although this phenomenon may be caused by an immune reaction to benign nevi for an unknown etiology, particularly in young patients, it has also been reported in patients with melanoma with and without treatment with PD-1 inhibitors [4]. The histology of a halo nevus is characterized by a dense, often lichenoid, lymphocytic infiltrate. Melanocytes may demonstrate reactive changes with rare mitoses superficially but should show evidence of maturation with descent in the dermis [4]. Changes indicative of melanoma, including significant cytologic atypia of melanocytes, pagetoid spread, mitoses in superficial and deep melanocytes, and lack of maturation, should not be present in halo nevi or nevi with reactive changes to immunotherapy. Regression of melanocytic nevi (MN) without a clinical halo, as seen in our patient, is not well documented [8]. The composition of the lymphocytic inflammatory infiltrate, along with the distribution of CD4+ and CD8+ T cells, may explain the varying clinical presentations [8]. The inflammatory response observed in MN during nivolumab therapy may result from antigenic overlap between melanocytes in benign nevi and melanoma cells [8].

The therapeutic agents our patient was treated with, nivolumab and ipilimumab, inhibit PD-1 and CTLA-4 signalling, respectively [6]. The mechanism of their inflammatory side-effect profile, while still under investigation, is thought to involve the activation of pro-inflammatory T cells (CD4+) and the loss of inhibitory function by T-regs [9]. While therapeutic in their purpose, this cancer-evading strategy is associated with IRAEs that may lead to widespread inflammatory damage [6].

Although our patient’s secondary lesions clinically presented without a halo, they all showed histologic features similar to those of halo nevi. The lesions were characterized by melanocytes surrounded by a marked inflammatory cell infiltrate. Mitoses were identified, but a double stain for Melan-A and Ki-67 helped determine that they were primarily present in inflammatory cells as opposed to melanocytes. Stains for SOX10 highlighted melanocytes primarily at the dermoepidermal junction in nests without prominent pagetoid spread and overall minimal architectural atypia, consistent with a benign process, and dermal melanocytes demonstrated maturation. Furthermore, p16 was retained in all the lesions, and PRAME was negative or focal and weak. Studies have shown that loss of p16 is more common in melanomas than in benign nevi [10]. PRAME is a newer marker with a staining pattern that is strongly and diffusely positive in most melanomas, while negative or weak in melanocytic lesions [11]. These differentiating histopathologic findings further supported that the pigmented lesions biopsied were benign with secondary reactive changes due to the immunotherapy.

The adverse effects associated with immune checkpoint inhibitors are dose-dependent and may arise after a certain threshold of molecules has been inhibited [6]. This would explain why some patients develop IRAEs and some do not [6]. In the present case, and in many cases of advanced stage melanoma, immunotherapy with both agents is started adjunctively, making distinguishing the side effect profile between the two difficult to determine. While adverse effects may occur, immune checkpoint therapy has been associated with improved survival among patients with melanoma, including this case [12].

Evolution of melanocytic nevi has emerged as a secondary effect of immunotherapy [2]. Continued monitoring of patients with a history of melanoma and further understanding of IRAEs is imperative. Monitoring with digital dermoscopy and total body photography may aid in additional surveillance efforts in all patients with melanoma to identify and differentiate secondary lesions [2]. Documentation of pigmented lesions prior to initiation of immunotherapy may also be helpful.

## Conclusions

This case illustrates the importance of interdisciplinary care in patients undergoing therapy for melanoma. The approach to this patient involving primary care, dermatology, oncology, and pathology teams showcases the importance of collaborative care, communication, and close monitoring for adverse reactions to therapeutic agents. Awareness of cutaneous IRAEs and the corresponding histopathologic changes facilitates accurate diagnosis and appropriate patient care.
